# Supplementary material for: Vesicles Shed by Pathological Murine Adipocytes Spread Pathology: Characterization and Functional Role of Insulin Resistant/Hypertrophied Adiposomes
Source: Int J Mol Sci. 2020 Mar 24;21(6):2252. doi: 10.3390/ijms21062252 (PMC7139903; doi:10.3390/ijms21062252)
Supplement: Supplementary file 1 [file ijms-21-02252-s001.zip › SUPPLEMENTARY METHODS.docx]

Vesicles shed by pathological murine adipocytes spread pathology: characterization and functional role of insulin resistant/hypertrophyated adiposomes

**CaminoTamara^1^, Lago-Baameiro N^1^, Bravo Susana Belén^2^, Sueiro Aurelio^3,4^, Couto Iván^1,4^, Santos Fernando^1,5^, Baltar Javier^1,5^, Casanueva F Felipe^3,6^, Pardo María*^1,6^.**

**SUPPLEMENTARY METHODS**

- 1. **PROTEIN IDENTIFICATION BY LC-MS/MS**

In order to make global protein identification, vesicles from an equal amount of all cell model secretomes [n=4 independent experiments; 3 replicates for each experiment: n=12] was loaded on a 10% SDS-PAGE gel. The run was stopped as soon as the front had penetrated 3 mm into the resolving gel.^1^ The protein band was detected by Sypro-Ruby fluorescent staining (Lonza), excised, and processed for in-gel, manual tryptic digestion as described elsewhere^2^. Peptides were extracted by carrying out three 20-min incubations in 40 μL of 60% acetonitrile dissolved in 0.5% HCOOH. The resulting peptide extracts were pooled, concentrated in a SpeedVac, and stored at −20 °C.

**QUALITATIVE-DDA MASS SPECTROMETRIC ANALYSIS**

Digested peptides were separated using Reverse Phase Chromatography. Gradient was created using a micro liquid chromatography system (Eksigent Technologies nanoLC 400, SCIEX) coupled to high speed Triple TOF 6600 mass spectrometer (SCIEX) with a micro flow source. The chosen analytical column was a silica-based reversed phase column YMC-TRIART C18 150 × 0.30 mm, 3 mm particle size and 120 Å pore size (YMC Technologies, Teknokroma). The trap column was a YMC-TRIART C18 (YMC Technologies, Teknokroma with a 3 mm particle size and 120 Å pore size, switched on-line with the analytical column. The loading pump delivered a solution of 0.1% formic acid in water at 10 µl/min. The micro-pump generated a flow-rate of 5 µl/min and was operated under gradient elution conditions, using 0.1% formic acid in water as mobile phase A, and 0.1% formic acid in acetonitrile as mobile phase B. Peptides were separated using a 90 minutes gradient ranging from 2% to 90% mobile phase B (mobile phase A: 2% acetonitrile, 0.1% formic acid; mobile phase B: 100% acetonitrile, 0.1% formic acid). Injection volume was 4 µl.

Data acquisition was performed in a TripleTOF 6600 System (SCIEX, Foster City, CA) using a Data dependent workflow. Source and interface conditions were the following: ionspray voltage floating (ISVF) 5500 V, curtain gas (CUR) 25, collision energy (CE) 10 and ion source gas 1 (GS1) 25. Instrument was operated with Analyst TF 1.7.1 software (SCIEX, USA). Switching criteria was set to ions greater than mass to charge ratio (m/z) 350 and smaller than m/z 1400 with charge state of 2–5, mass tolerance 250ppm and an abundance threshold of more than 200 counts (cps). Former target ions were excluded for 15 s. The instrument was automatically calibrated every 4 hours using as external calibrant tryptic peptides from PepCalMix (SCIEX, USA).

**QUANTITIVE-DIA SWATH (Sequential Window Acquisition of All Theoretical Mass Spectra) ANALYSIS**

***Creation of the spectral library for SWATH analysis***

To build the MS/MS spectral libraries, the peptide solutions were analyzed by the previous shotgun data-dependent acquisition (DDA) approach by micro-LC-MS/MS. To get a good representation of the peptides and proteins present in all samples, pooled vials of the 3 biological samples were prepared using equal mixtures of the original samples. Four µg of each pool was separated into a micro-LC system Ekspert nLC425 (Eksigen, Dublin, CA, USA) using an YCM-TriartC18 column (150μm × 0.3mm, 12 nmm, s-3m) (YMC CO.Japan) at a flow rate of 5L/min. Water and ACN, both with 0.1% formic acid, were used as solvents A and B, respectively. The gradient run consisted of 5% to 95% B for 30 min, 5 min at 90% B and finally 5 min at 5% B for column equilibration, for a total run time of 40 min. A micro source (SCIEX, USA) was used for the interface between microLC and MS [TripleTOF 6600 System (SCIEX, Foster City, CA)], with an application of 2600 V voltage. The acquisition mode consisted of a 250 ms survey MS scan from 400 to 1250 m/z followed by an MS/MS scan from 100 to 1500 m/z (25 ms acquisition time) of the top 100 precursor ions from the survey scan, for a total cycle time of 2.8 s. The fragmented precursors were then added to a dynamic exclusion list for 15 s to avoid single charged ions to be included to the MS/MS analysis.

The peptide and protein identifications to perform the SWATH library, were made by means of Protein Pilot software (version 5.0.1, SCIEX) using a Human specific Uniprot database (release January 2018), specifying iodoacetamide as Cys alkylation. The false discovery rate (FDR) was set to 1 for peptides and proteins. The MS/MS spectra of the identified peptides were later used to generate the spectral library for SWATH peak extraction utilizing PeakView Software (version 2.2, (SCIEX) with SWATH Acquisition MicroApp (version 2.0, (SCIEX). Peptides with a confidence score above 99% were incorporated in the spectral library.

***Relative quantification by SWATH acquisition***

Samples from all cell models secreted vesicles of 4 independent experiments were analyzed using a data-independent acquisition (DIA) method making 3 technical replicates for each sample (n=12 samples from each cell model). Each sample was analyzed using the LC-MS/MS equipment and LC gradient described above for building the spectral library but using instead the SWATH-MS acquisition method. The method comprised repeating a cycle that makes 65 TOF MS/MS scans (400 to 1500 m/z, high sensitivity mode, 50 ms acquisition time) of overlapping sequential precursor isolation windows of variable width (1 m/z overlap) covering the 400 to 1250 m/z mass range with a previous TOF MS scan (400 to 1500 m/z, 50 ms acquisition time) for each cycle. Total cycle time was 6.3 s. For every sample set, the width of the 65 variable windows was optimized as stated by the ion density found in the DDA runs using a SWATH variable window calculator worksheet from SCIEX.

**Data analysis**

To achieve the global proteome analysis data files were processed using ProteinPilot^TM^ 5.0.1 software from SCIEX which uses the algorithm ParagonTM for database search and Progroup^TM^ for data grouping. Data were searched using a Human specific Uniprot database (released January 2018). False discovery rate was performed using a non lineal fitting method displaying only those results that reported a 1% Global false discovery rate or better. ^3^.

To make the SWATH quantitative analysis, the targeted data extraction of the fragment ion chromatogram traces was performed by PeakView (version 2.2) using the SWATH Acquisition MicroApp (version 2.0). This application processed the data by means of the spectral library created from the DDA analysis. Up to ten peptides per protein and seven fragments per peptide were selected, based on signal intensity; any shared and modified peptides were removed from the processing. Five minute windows and 50 ppm widths were used to extract the ion chromatograms. The retention times from the peptides that were selected for each protein were realigned in each run according to the calibration curve retention time (iRT). The extracted ion chromatograms were then created for each selected fragment ion; the peak areas for the peptides were established by summing the peak areas from the corresponding fragment ions. PeakView computed an FDR and a score for each assigned peptide in relation to the chromatographic and spectra components; only those peptides with an FDR below 1% were elected for protein quantification. Protein quantification was calculated by adding the peak areas of the corresponding peptides. The integrated peak areas (processed .mrkvw files from PeakView) were directly exported to the MarkerView software (SCIEX) for relative quantitative analysis as previously described ^4^. The export generates three files containing quantitative information about individual ions, the summed intensity of different ions for a particular peptide and the summed intensity of different peptides for a particular protein. Data alignment by MarkerView compensates for minor variations in both mass and retention time values, ensuring that identical compounds in different samples are accurately compared to one another. To control for possible uneven sample loss across the different samples during the sample preparation process, we performed a global normalization based on the total sum of all the peak areas extracted from all the peptides and transitions across the replicates of each sample. The average MS peak area of each protein from 4 independent samples and 3 technical replicates for each sample (n=12 from each cell model) was derived from the SWATH-MS of each sample followed by Student’s *t*-test analysis using the MarkerView software for comparison among the samples based on the averaged area sums of all the transitions derived for each protein. The *t*-test will indicate how well each variable distinguishes the two groups, reported as a *p*-value. For each library, its set of differentially expressed proteins (*p*-value *<*0.05) with a 1.5 fold in- or decrease was selected.

1 Bonzon-Kulichenko E, Pérez-Hernández D, Núñez E, Martínez-Acedo P, Navarro P, Trevisan-Herraz M *et al.* A robust method for quantitative high-throughput analysis of proteomes by 18O labeling. *Mol Cell Proteomics* 2011; **10**: M110.003335.

2 Shevchenko A, Wilm M, Vorm O, Jensen ON, Podtelejnikov A V, Neubauer G *et al.* A strategy for identifying gel-separated proteins in sequence databases by MS alone. *Biochem Soc Trans* 1996; **24**: 893–896.

3 Shilov I V., Seymour SL, Patel AA, Loboda A, Tang WH, Keating SP *et al.* The Paragon Algorithm, a Next Generation Search Engine That Uses Sequence Temperature Values and Feature Probabilities to Identify Peptides from Tandem Mass Spectra. *Mol Cell Proteomics* 2007; **6**: 1638–1655.

4 Meyer JG, Schilling B. Clinical applications of quantitative proteomics using targeted and untargeted data-independent acquisition techniques. *Expert Rev Proteomics* 2017; **14**: 419–429.
